# Characterization of ENU-induced Mutations in Red Blood Cell Structural Proteins

**DOI:** 10.5936/csbj.201303012

**Published:** 2013-09-23

**Authors:** Katrina Kildey, Robert L. Flower, Thu V. Tran, Robert Tunningley, Jonathan Harris, Melinda M. Dean

**Affiliations:** a Research and Development, Australian Red Cross Blood Service, Brisbane, Australia; b Queensland University of Technology, Brisbane, Australia; c Australian Phenomics Facility, Canberra, Australia

**Keywords:** Red Blood Cell, N-ethyl-N-nitrosourea (ENU), Ankyrin, Spectrin-1β, Band 4.1, Missense Library

## Abstract

Murine models with modified gene function as a result of N-ethyl-N-nitrosourea (ENU) mutagenesis have been used to study phenotypes resulting from genetic change. This study investigated genetic factors associated with red blood cell (RBC) physiology and structural integrity that may impact on blood component storage and transfusion outcome. Forward and reverse genetic approaches were employed with pedigrees of ENU-treated mice using a homozygous recessive breeding strategy. In a “forward genetic” approach, pedigree selection was based upon identification of an altered phenotype followed by exome sequencing to identify a causative mutation. In a second strategy, a “reverse genetic” approach based on selection of pedigrees with mutations in genes of interest was utilised and, following breeding to homozygosity, phenotype assessed. Thirty-three pedigrees were screened by the forward genetic approach. One pedigree demonstrated reticulocytosis, microcytic anaemia and thrombocytosis. Exome sequencing revealed a novel single nucleotide variation (SNV) in *Ank1* encoding the RBC structural protein ankyrin-1 and the pedigree was designated Ank1^EX34^. The reticulocytosis and microcytic anaemia observed in the Ank1^EX34^ pedigree were similar to clinical features of hereditary spherocytosis in humans. For the reverse genetic approach three pedigrees with different point mutations in *Spnb1* encoding RBC protein spectrin-1β, and one pedigree with a mutation in *Epb4.1*, encoding band 4.1 were selected for study. When bred to homozygosity two of the spectrin-1β pedigrees (a, b) demonstrated increased RBC count, haemoglobin (Hb) and haematocrit (HCT). The third *Spnb1* mutation (spectrin-1β c) and mutation in *Epb4.1* (band 4.1) did not significantly affect the haematological phenotype, despite these two mutations having a PolyPhen score predicting the mutation may be damaging. Exome sequencing allows rapid identification of causative mutations and development of databases of mutations predicted to be disruptive. These tools require further refinement but provide new approaches to the study of genetically defined changes that may impact on blood component storage and transfusion outcome.

## Introduction

Red blood cell transfusion remains an essential medical therapy for the treatment of a broad range of anaemias. Currently packed red blood cells (PRBCs) for clinical transfusion have a 42 day expiry post-collection. During storage red blood cells (RBC) undergo numerous biophysical and biochemical changes [1, 2] (termed the storage lesion) that may alter the biological function of the transfused RBC and/or the immune response of patients post-transfusion. Clinical studies have reported an association between RBC storage and increased patient morbidity post-transfusion [3–5]. In addition, there is evidence of donor variability in the lifespan of RBCs in storage and onset of the storage lesion [6].

There are two alternative approaches for investigation of association of the product of a genetic open reading frame to a phenotype, referred to as reverse and forward genetics. The “reverse genetic” approach investigates the function of a known gene by phenotypic analysis of cells or organisms in which the function of this gene is impaired [7]. In contrast, the “forward genetic” approach is a tool to identify modifications in cells or organisms through phenotypic analysis, then determine the genetic basis of an altered phenotype [8]. In this study, we have investigated both the forward and reverse approaches to study single nucleotide variation (SNV)-based changes in proteins critical for RBC function that may impact on blood component storage and transfusion outcomes using the N-ethyl-N-nitrosourea (ENU)-mutagenised murine model.

ENU mutagenesis is a technique utilised to produce new pedigrees of mice with SNVs that facilitate investigation of protein function and disease states. ENU is a DNA-alkylating agent that introduces a high rate of random genome-wide SNVs into the mouse germline by affecting the spermatogonial (premeiotic) germ cells in the mice testes [9]. Importantly, the phenotypes from these point mutations more closely model human genetic variation than mouse transgenic models, as 70% of the disease causing alleles so far described in humans are the result of SNVs [10]. SNVs are more likely to result in inactive or altered function of a particular domain of a protein, highlighting active sites or functions of variant forms of the protein. ENU-mutagenised mice are available for selection from a number of libraries for investigation of a defined gene mutation (reverse genetics). In addition new gene function and potential disease association can be discovered by screening ENU pedigrees for phenotypes of interest (forward genetics).

Mature human RBC's have a biconcave discoid shape and can survive shearing stress and undergo elastic deformation to allow passage through capillaries. The elastic filamentous RBC cytoskeleton is composed of four major interconnected components: spectrin1-α (240 kDa), spectrin1-β (220 kDa), band 4.1 (80 kDa) and actin (β-type; 42 kDa), which are the basis of the RBC's structure and mechanical properties [11]. The RBC cytoskeletal proteins are attached to the plasma membrane through interaction with ankyrin and transmembrane protein band 3 that is embedded in the lipid bilayer [12–14]. Disruptions to these protein interactions have been associated with genetically-inherited RBC-related diseases in human populations including hereditary spherocytosis (HS), elliptocytosis, stomatocytosis and pyropoikilocytosis [15–17]. In addition to effects on RBC morphology and structural integrity, disruptions in RBC cytoskeletal proteins have been reported to impact on RBC survival and/or differentiation [18, 19]. Human and murine RBCs share a similar cytoskeletal structure [20], though they differ somewhat in lifespan (human RBC predicted 120 days, murine RBC estimated 50 days) in circulation [21]. Murine RBC have been used to model human RBC-related disease [22–24] and provide a suitable platform to study genetic changes in RBCs that may impact on the structural integrity, differentiation and survival of RBCs and therefore component storage and transfusion outcomes.

In this study, we have used murine ENU-mutated models, using both the forward and reverse genetic approaches, to investigate the effect of SNV-based changes on RBC. Pedigrees with SNVs in *Epb4.1* (encoding band 4.1), and three different SNVs in *Spnb-1* (encoding spectrin-1β), selected for the reverse genetic approach, demonstrated some perturbation of haematological parameters. In contrast, the forward genetic approach resulted in the generation of a pedigree characterised by reticulocytosis, microcytic anaemia and thrombocytosis. This phenotype was determined to be the result of a novel mutation in *Ank1*, encoding the RBC structural protein ankyrin-1.

## Experimental procedures

### ENU-Mutagenesis

ENU-mutagenesis was performed on C57BL/6 mice by the Australian Phenomics Facility (Australian National University (ANU) Canberra) [9, 25]. Briefly, male C57BL/6 mice were treated with three intraperitoneal injections of 80 µg ENU/gram of body weight. Ten weeks post mutagenesis ENU-treated males were crossed with multiple female C57BL/6 mice to produce pedigrees with randomly incorporated point mutations. ENU-pedigrees on a C57BL/6 genetic background were produced using a three generation approach to reveal recessive mutations [9]. To reduce the mutation load in the resultant pedigrees G1 mice were crossed with background C57BL/6 mice, and G2 siblings mated (or G2 backcrossed to G1) to generate homozygous G3 mice. Mice were maintained at the Australian Phenomics Facility, Canberra. Animal protocols were reviewed and approved by the Australian National University experimentation ethics committee, and all procedures were carried out under the guidelines of the Australian code of practice for the care and use of animals for scientific purposes.

### Pedigree screening for altered haematological phenotype (forward approach)

Using a forward genetics approach an ENU-induced mutant pedigree was selected for further breeding and analysis based on an identifiable phenotype. Briefly, using a “homozygote recessive” breeding strategy, blood from 33 ENU-induced mutant pedigrees was screened for evidence of a changed blood picture by a differential cell count (RBC, platelet, white blood cell (WBC), neutrophil, lymphocyte, monocyte, eosinophil, basophil, and reticulocytes) and measurement of standard haematological parameters (Hb, HCT, and mean corpuscular volume (MCV)) undertaken using an ADVIA analyser (Siemens Healthcare Global). Mice with altered haematological parameters were selectively bred. Causative mutations were identified via exome sequencing (G4 mice). Where indicated mice were sacrificed, spleens excised and measured (wet weight (g), length (cm)).

### Selection of ENU-pedigrees from missense mutation library (reverse approach)

Based on the presence of ENU-induced mutation in RBC structural proteins, four pedigrees were selected from the Australian Phenomics Facility missense mutation library [26]. Three pedigrees, each with different SNV in *Spnb1* (encoding spectrin-1β), and one pedigree with a SNV in *Epb4.1* (encoding band 4.1) were selected for assessment in a “reverse genetics” approach to investigate factors that may alter RBC integrity (Table 1]. Homozygous pedigrees for the *Epb4.1* SNV and the three individual *Spnb1* SNVs were generated from G2 heterozygous mice using genetic screening. All genomic positions of the mutations were referenced to the Genome Reference Consortium Mouse Build 38 (GRCm38).

**Table 1:** Pedigrees selected for mutations in RBC structural genes.

Gene (Protein)	Chromosome	Base change	Amino acid change	Amino acid position	Mutation genomic position (bp)	Domain	PolyPhen score
*Spnb1*(Spectrin-1β) (a)	12	A→G	S→P	857	76,620,753	Spectrin-binding	0.24 benign
*Spnb1*(Spectrin-1β) (b)	12	T→C	D→G	664	76,621,331	Spectrin-binding	0.2 benign
*Spnb1*(Spectrin-1β) (c)	12	T→C	K→E	462	76,623,169	Spectrin-binding	1 probably damaging
*Epb4.1*(Band 4.1)	10	A→G	T→A	669	25,501,836	hydrophilic	0.77 possibly damaging

### Exome sequencing

ENU variations were identified via exome sequencing as previously described [25]. Briefly, exome enriched, paired end libraries were prepared from genomic DNA of each sample. The Illumina paired-end genomic DNA sample preparation kit (PE-102-1001, Illumina) was used for preparing the libraries including end repair, A-tailing and ligation of the Illumina adaptors. Nimblegen SeqCap baits (#999042611, Roche Diagnostics) designed to 203,225 exons and 54Mb of the reference coding regions were used to enrich for the mouse exome. Each sample was prepared with an index using the Illumina multiplexing sample preparation oligonucleotide kit (PE-400-1001, Illumina) and then pooled in batches of four in equimolar amounts prior to capture. Each capture was sequenced in a single lane of a HiSeq 2000 (Illumina) as 100bp paired end libraries. Sequence reads were mapped to the mouse reference assembly and SNVs identified and annotated [23].

### SNV genotyping

Primers were designed using Amplifluor™ Assay Architect™ on-line design program (Table 2). Multiplexed PCR was performed in 96 well plates on Eppendorf MasterCycler ProS thermocycler (POCD Pty Ltd, Artarmon, NSW). Genomic DNA (gDNA) was obtained from 2-3 mm ear punch. Sample was prepared by adding the ear tissue to 15 µl of TE-Tween Lysis buffer (50 mM Tris HCl, pH 8.0, 0.125 mM EDTA, 2% Tween 20) and sample centrifuged (2 min, 2500 g). The prepared tissue sample was digested in 15 µl ear punch digestion mix (14 µl TE-Tween Lysis Buffer, 1.0 µl Proteinase K (20 mg/ml)), by incubating at 55°C for 1 hour, followed by 99°C for 10 min to denature the proteinase K. Samples were centrifuged (10 min, 2500 g) and washed with 180 µl of sterile ddH_2_O.

**Table 2:** Forward and reverse primers utilised for genotyping ENU-induced mutations in mice.

Gene	Genotype	Forward primer	Reverse Primer
*Spnb1* (a)	Variant	GAAGGTCGGAGTCAACGGATTTACACCGTGTTTGGGGAGC	TCATCCACAGCTCACAGGCAT
	Reference	GAAGGTGACCAAGTTCATGCTGTACACCGTGTTTGGGGAGT	
*Spnb1* (b)	Variant	GAAGGTGACCAAGTTCATGCTCAGGTCCTTGCCGTAGC	TCTGGAAGTTCTTCTGGGAGAT
	Reference	GAAGGTCGGAGTCAACGGATTGTCAGGTCCTTGCCGTAGT	
*Spnb1* (c)	Variant	GAAGGTCGGAGTCAACGGATTTCTCAATGGCTTCGTGCTTCTC	GGGCAGGATAACTTTGGGTA
	Reference	GAAGGTGACCAAGTTCATGCTGTCTCAATGGCTTCGTGCTTCTT	
*Epb4.1*	Variant	GAAGGTCGGAGTCAACGGATTTTCTCCTCCACTACATTCAGAGC	TTTCCCTCATCCGTCTTGTTTT
	Reference	GAAGGTGACCAAGTTCATGCTTCTCCTCCACTACATTCAGAGT	
*Ank1*	Variant	GAAGGTGACCAAGTTCATGCTACATTGGCAGGTGGCAGCTCAT	ACGAGGACACACAGCACAT
	Reference	GAAGGTCGGAGTCAACGGATTATTGGCAGGTGGCAGCTCAC	

PCR was performed in a 5 µl reaction containing 1 µl of gDNA, 0.1 µM PCR Amplifluor primer mix, 0.25 µM amplifluor universal FAM Primer (IDT), 0.25 µM amplifluor universal JOE Primer (IDT), 0.2 mM dNTPs each, 10x Taq DNA Polymerase PCR buffer (Invitrogen), 1.8 mM MgCl_2_, and 0.05 units of MyTaq™ HS DNA Polymerase. PCR cycling conditions were 95°C for 4 min, followed by 35 cycles of 95°C for 10s, 60°C for 20s and 72°C for 10s followed by a final elongation of 72°C for 3 min.

### Bioinformatics modelling

To further annotate the SNVs bioinformatics modelling was utilised to evaluate and predict the functional significance of mutations for each pedigree. A polymorphism phenotyping computational tool (PolyPhen-2) predicting potential impact of amino acid substitutions on the structure and function of the protein (8 sequence based and 3 structure based) was utilised [24]. The Polyphen-2 software was adjusted to more accurately predict the damaging effect of SNVs in the mouse genome [23]. PolyPhen score and predictive disruption indicated in Table 1.

### Eosin-5-maleimide (E5M) staining of RBC cytoskeleton

E5M binds to band 3 and Rh-related proteins on RBCs and is utilised as a tool to assess changes in RBC surface area. E5M staining was based on a previously published assay [27]. Briefly, 0.5 mg/ml E5M was prepared in PBS. Ten microliters E5M (0.5 mg/ml) was mixed with 5µL of pre-washed murine RBC (PBS pH 7.4) and incubated (room temperature (RT), dark, 1 hour) with intermittent mixing. RBCs were washed thrice with 500 µl PBS. On final wash the cell pellet was resuspended in 500 µl PBS. The labelled cell suspension was further diluted 1:10 in PBS for analysis via flow cytometry (BD FACSCanto II, 3 laser flow cytometer, BD Biosciences, USA).

### Osmotic fragility

Differences in the osmotic fragility of murine RBC were evaluated by lysis in hypotonic saline, based on a previously published assay with some minor modifications [28]. Briefly, eleven hypotonic-saline solutions (0.9, 0.8, 0.75, 0.7, 0.65, 0.6, 0.55, 0.5, 0.45, 0.4, and 0.3% NaCl respectively) were prepared in milli-Q H_2_O. In a v-bottom 96 well microtitre plate, 2 µl of murine whole blood (representing approx. 2x10^7^ cells) was added followed by 200 µl of each saline solution or H_2_O (100% lysis control), and incubated (30 min, RT). After incubation, plates were centrifuged (1000xg, 5 min) and 100 µl of the supernatant was transferred to a fresh 96 well flat bottom microtitre plate. Haemolysis was measured at 450nm.

### Quantification of IL-6 and RANTES via cytometric bead array (CBA)

Murine plasma was incubated with analyte-specific bead populations, and bound cytokine and chemokine analytes were detected with PE-conjugated antibody. Inflammatory mediators interleukin-6 (IL-6) and RANTES (CCL5), were quantified using Becton Dickenson (BD) CBA (BD Biosciences, Australia) according to the manufacturer's instructions with minor modifications [29]. In brief, plasma was diluted 1:4 and incubated with 0.5µL of each capture bead for 1hr; 0.5µL of each detection reagent was then added and incubated for a further 2hr and analysed on a BD FACSCanto II (BD Biosciences, Australia). FACSArray software (BD Biosciences, CA, USA) was used for analysis to quantify concentrations from the standard curves run in parallel. Standard curves for IL-6 and RANTES assays ranged from 10-2500 pg/ml.

### Quantification of erythropoietin and thrombopoietin via ELISA

Where indicated, levels of erythropoietin (EPO) and thrombopoietin (TPO) were assessed via ELISA according to manufactures instructions (MEP00B, MTP00; R&D systems, USA). Briefly, plasma was diluted 1:2 (TPO) or 1:5 (EPO) in respective assay diluents and incubated for 2hrs. Plates were washed and respective conjugate antibody added for 2hrs. Plates were washed before addition of substrate for 30min and reaction stopped with diluted hydrochloric acid. Absorbance measured at 450nm (540nm reference).

### Statistical Analysis

Unpaired data was compared using a t-test. One-Way Analysis of Variance (ANOVA) was performed where indicated. GraphPad Prism (V) was used for all statistical analysis with 95% CI (GraphPad Software, La Jolla, USA).

## Results

### Identification of a novel ENU-pedigree with elevated reticulocyte count

**Figure 1:**
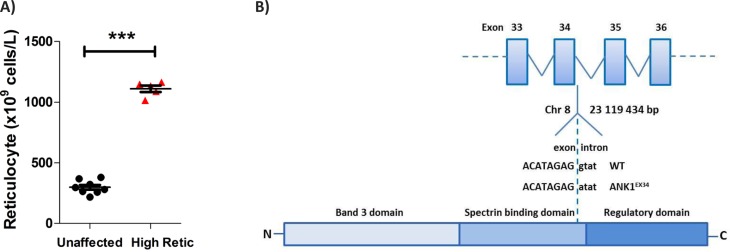
**Identification of novel high reticulocyte ENU phenotype and identification of causative mutation**. **A)** G2 ENU treated mice were screened for key haematological parameters. A phenotype characterised by high reticulocyte was identified (*** p < 0.0001 affected *cf*. unaffected). Affected mice were selected for further breeding and analysis. **B)** Schematic representation of the position of the ENU-induced mutation in *Ank1*. Sequencing revealed the phenotype was a result of a novel splice variant in *Ank1*, specifically a G → A substitution at the first base of the donor splice site for exon 34.

For the forward genetic approach, 33 ENU-induced mutant pedigrees were screened for evidence of a changed blood picture. Screening resulted in identification of a subset of mice with an affected phenotype characterised by a significant elevation in reticulocyte count (p < 0.0001 unpaired T-test affected cf. unaffected mice; Figure 1A). The affected mice were chosen for further breeding and analysis.

### Identification of a novel mutation in RBC cytoskeleton protein ankyrin-1

Exome sequencing of G4 affected mice identified a SNV in *Ank1* (encoding RBC structural protein ankyrin-1) as a candidate ENU-induced mutation resulting in the high reticulocyte phenotype. Sequencing revealed a G→A substitution on Chromosome 8 (base pair position 23,119,434: GRCm38); the first base of the splice donor site 5’ of *Ank1* exon 34. The *Ank1* mutation was predicted to result in a splice variant of ankyrin-1 with a potential loss of the amino acids encoded for by exon 34 (Figure 1B). Ankyrin-1 is an erythrocyte cytoskeletal protein that provides a link between RBC membrane structural proteins (spectrin and band 3) and the inner surface of the lipid bilayer [14, 30]. The mutation reported here is different from previously reported *Ank1* mutations in both humans [15, 31] and mice [19, 32–34]. Defects in ankyrin-1 have been found in >50% of cases of HS in humans, characterised by evidence of haemolysis with anaemia, spherocytic RBC, reticulocytosis, jaundice, gallstones and splenomegaly [35]. This novel ankyrin pedigree may model some forms of human HS and is a valuable tool for further characterisation of this disease. The pedigree was designated Ank1^EX34^.

### Investigation of haematological parameters in Ank1^EX34^, band 4.1 and spectrin-1β pedigrees

In a reverse genetics approach, four ENU pedigrees with SNV in genes encoding RBC structural proteins (band 4.1 and three individual spectrin-1β (designated a, b, c)) were selected from the Australian Phenomics Facility missense mutation library [26]. Together with the novel Ank1^EX34^ pedigree differential cell counts and standard haematological parameters were assessed (Table 3). In homozygous Ank1^EX34^ mice, the significantly elevated reticulocyte count was consistently observed (p < 0.0001), and was compounded with significantly decreased RBC count (p < 0.0001), Hb (p < 0.0001), HCT (p < 0.0001), and MCV (p < 0.0001). In addition, homozygous Ank1^EX34^ mice exhibited thrombocytosis as evidenced by significantly elevated platelet count (p < 0.0001: Table 3). Mice heterozygous for the *Ank1* mutation also showed a significantly increased reticulocyte count (p = 0.0026) and reduced MCV (p = 0.0114; Table 3).

**Table 3:** Differential cell counts and haematological parameters for ENU-mutant pedigrees [mean (SD)]. Whole blood from mice with homozygous (HOM) or heterozygous (HET) SNV in genes encoding RBC structural proteins were assessed for differences in haematological parameters using the ADVIA analyser. *p < 0.05 unpaired T-test *cf*. unaffected siblings.

Ankyrin-l					Spectrin-lβ (a)			Spectrin-lβ (b)			Spectrin-lβ (c)			Band 4.1		
		Hom n = 11	Het n = 11	Unaff n = 8	Hom n = 5	Het n = 7	Unaff# n = 5	Hom n = 5	Het n = 8	Unaff# n = 4	Hom n = 4	Het n = 3	Unar# n = 2	Hom n = 2	Het n = 7	Unafl# n = 8
Differential cell count	WBC (x10^3^ cellsVD	*8.4* (1-7)	5.7 (1.8)	6.5 (2.0)	6.4 (2.6)	8.9 (2.4)	5.5 (0.8)	8.3 (1.6)	7 (1.6)	7 (1.7)	8.2 (0.4)	**7** (1.3)	7.3 (0.7)	7.8 (1-6)	7.9 (1.7)	7.4 (2-2)
	RBC (x10^6^cells/µL)	7.2* (0.9)	11.6 (15)	10.97 (0.8)	12.1* (0.5)	11.4 (1-2)	10.3 (1.3)	12.2* (0.8)	10.9 (0.8)	10.9 (0.8)	11.6 (0-9)	9.9 (0.9)	10.7 (0.2)	10.4 (0.6)	10.3 (0.7)	10 (0.5)
	Reticulocyte (x10^3^cells/µL)	1111.0* (60.4)	428.1* (92.0)	298.7 (55.9)	342.8 (26.4)	296.3 (69.3)	348.0 (226.8)	390.1 (54.5)	298.3 (65.4)	349.5 (115.3)	291.7 (31.0)	294.2 (30.6)	278.8 (33.7)	265.1 (53.9)	255.7 (35.6)	292.5 (627)
	Platelet (x10^3^celb/µL)	2542* (618.5)	1373 (211.9)	1273 (232.3)	1298 (177)	1375 (267)	1164 (263)	1258 (148)	1219 (183)	1421 (151)	1311 (147)	1301 (243)	1230 (34)	1290 (93)	1201 (214)	1225 (150)
Haematological parameters	Hb (g/L)	127.2* (13-3)	181.1 (24.0)	186.8 (18.0)	210.6* (5.3)	199.6 (24.7)	179 (25.2)	200.4* (7.4)	189 (21.0)	182 (14.7)	201 (15.1)	184.7 (12.7)	188 (2.8)	181 (7.1)	180-9 (11.6)	175.8 (10.7)
	HCT	0.30* (0.04)	0.47 (0.06	0.5 (0.05)	0.55* (0.04)	0.52 (0.07)	0.47 (0.05)	0. 60* (0.04)	0.5 (0.05)	0.5 (0.05)	0.6 (0.04)	0.5 (0.05)	0.51 (0.01)	0,43 (0.01)	0.43 (0.03)	0.42 (0.02)
	MCV (fl)	41.6* (1.7)	40.7* (3.9)	45.6 (3.5)	45.3 (1.8)	45.6 (2.1)	15.6 (1-3)	46,4 (1.7)	48.3* (1.5)	45-9 (1.6)	47.7 (0.2)	48.8 (1.2)	47.3 (0.1)	41.4 (0.8)	41.4 (0.3)	11.8 (0.8)

# unaff refers to unaffected siblings in the pedigree

While mutation in *Ank1* predominantly resulted in reduction of key RBC parameters indicating a disruption in RBC structural integrity, SNV in *Spnb1* (a) and S *pnb1* (b) resulted in elevated RBC parameters. In mice carrying homozygous *Spnb1* (a) or *Spnb1* (b) mutation, significantly increased RBC count (p = 0.0211 (a), p = 0.0476 (b)), haemoglobin (p = 0.0253 (a), p = 0.0432 (b)), and HCT (p = 0.0186 (a), p = 0.0431 (b)) was evident (Table 3). With the exception of a significantly increased MCV (P = 0.0285) in heterozygous *Spnb1* (b) mice, these altered blood parameters were not evident in the heterozygous *Spnb1* (a) or (b) mice.

SNV in *Spnb1* (c) or *Epb4.1* did not significantly affect the haematological phenotype, as there was no significant difference between the blood picture in either homozygous or heterozygous mice with these mutations compared to their unaffected siblings (Table 3). Of note, the MCV and HCT was reduced in all mice studied from the band 4.1 pedigree, regardless of the carriage of *Epb4.1* mutation (unaffected, heterozygous or homozygous) and it is hypothesised that another causative mutation was responsible for the reduced red cell size in this pedigree.

### Increased osmotic fragility in Ank1^EX34^ pedigree

**Figure 2:**
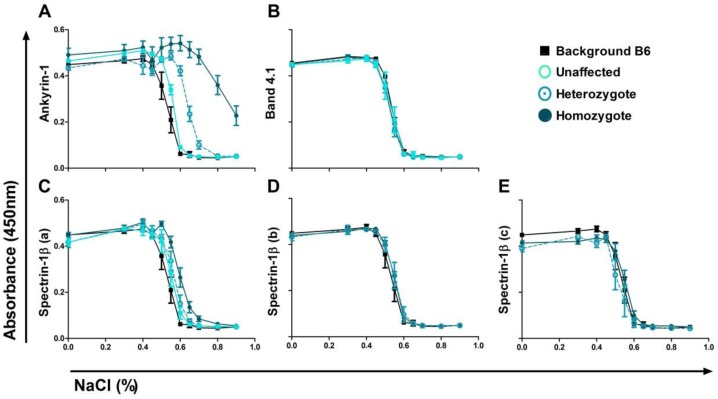
**Osmotic fragility of RBC from ENU pedigrees. A)** RBC from the ANK-1^EX34^ pedigree (ankyrin-1) had increased osmotic fragility (ANOVA p < 0.0001). Homozygous ANK-1^EX34^ had increased osmotic fragility compared to both the background and heterozygous mice (p < 0.001, p < 0.05 respectively; Tukey post-test). **B-E)** No difference in RBC osmotic fragility for band 4.1 or any of the spectrin-1β pedigrees.

Standard osmotic fragility testing was performed on red cells from all pedigrees to investigate potential changes in RBC structural integrity. Homozygous Ank1^EX34^ mice demonstrated significantly increased osmotic fragility (Figure 2A). There was no significant difference in the osmotic fragility of RBCs from mice from pedigrees either homozygous or heterozygous for the *Epb4.1* or any of the *Spnb1* mutations (Figure 2B-E).

### Reduced binding of E5M to RBC membrane in Ank1^EX34^

**Figure 3:**
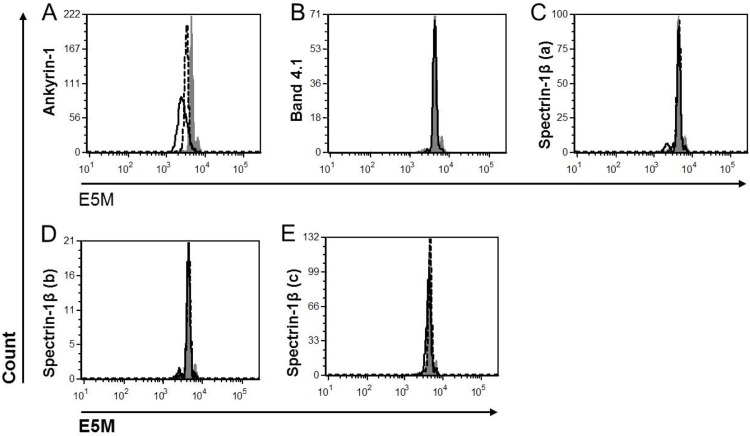
**Representative histograms of E5M flow cytometric analysis of RBC integrity**. Grey solid peaks indicate E5M on RBC from C57/B6 background mice, black solid line indicates homozygote RBC and dashed line heterozygote RBC. **A)** Ank1^EX34^ (Ankyrin-1) homozygotes displayed reduced E5M staining and heterozygotes an intermediate reduction in E5M staining. **B-E)** No differences observed in the band 4.1 or spectrin-1β pedigrees.

**Figure 4:**
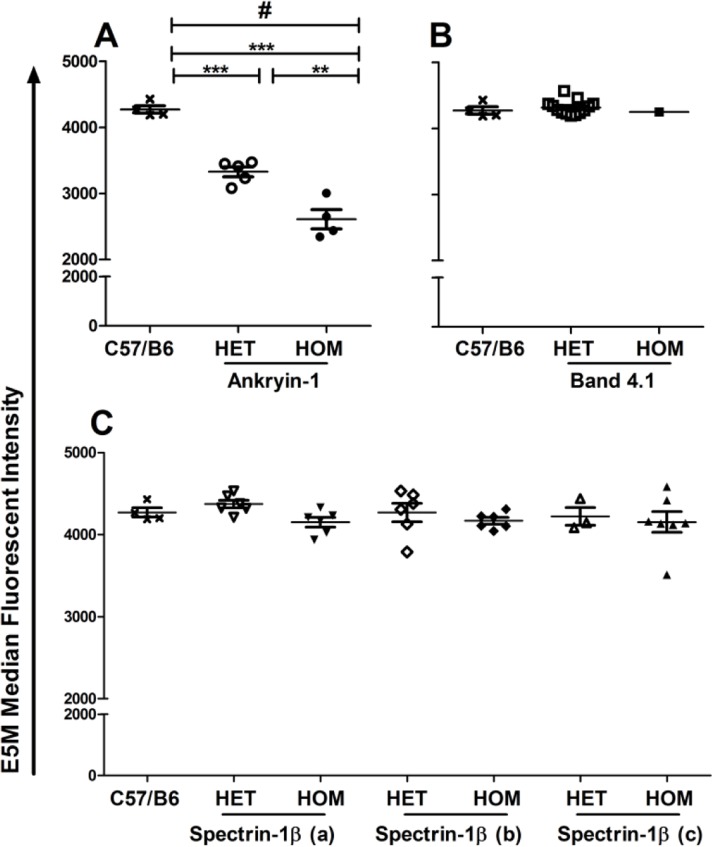
**Median fluorescent intensity (MFI) of E5M staining of RBC from ENU-pedigrees. A)** Ank1^**EX34**^ (Ankyrin-1) MFI was reduced in both the homozygotes (HOM) (p < 0.0001) and heterozygotes (HET) (p < 0.001) cf. C57/B6 background pedigree. No difference in RBC integrity was observed for **B)** band 4.1 or **C)** spectrin-1β pedigrees. # ANOVA; **p < 0.01, ** p < 0.001, Tukeys post-test

E5M binds to band 3 (at lysine 430) and Rh-related proteins on RBC membranes; a reduction in E5M staining indicates a disruption in the RBC cytoskeleton [36]. A clinical diagnosis of HS is associated with reduced E5M binding to the RBC membrane [27, 36–38]. As expected the Ank1^EX34^ pedigree demonstrated significantly reduced E5M binding (Figure 3A). This reduction was evident in both heterozygous and homozygous mice, though the effect was more pronounced in the homozygous mice as indicated by fluorescent intensity (Figure 4A). There was no difference in E5M staining of RBCs from the spectrin-1β (a), spectrin-1β (b), spectrin-1β (c), or band 4.1 pedigrees (Figure 3B-E, Figure 4B-C).

### Ank1^EX34^ pedigree had enlarged spleens and modulation of RBC and platelet growth factors

**Figure 5:**
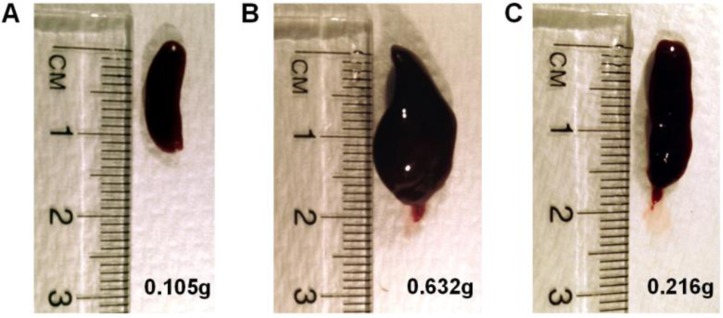
**Excised spleens from Ank1** ^**EX34**^ **pedigree. A)** Spleen of unaffected Ank1^EX34^ sibling. **B & C)** Spleens from Ank1^EX34^ homozygotes. Weights of spleens indicated in grams (g).

**Figure 6:**
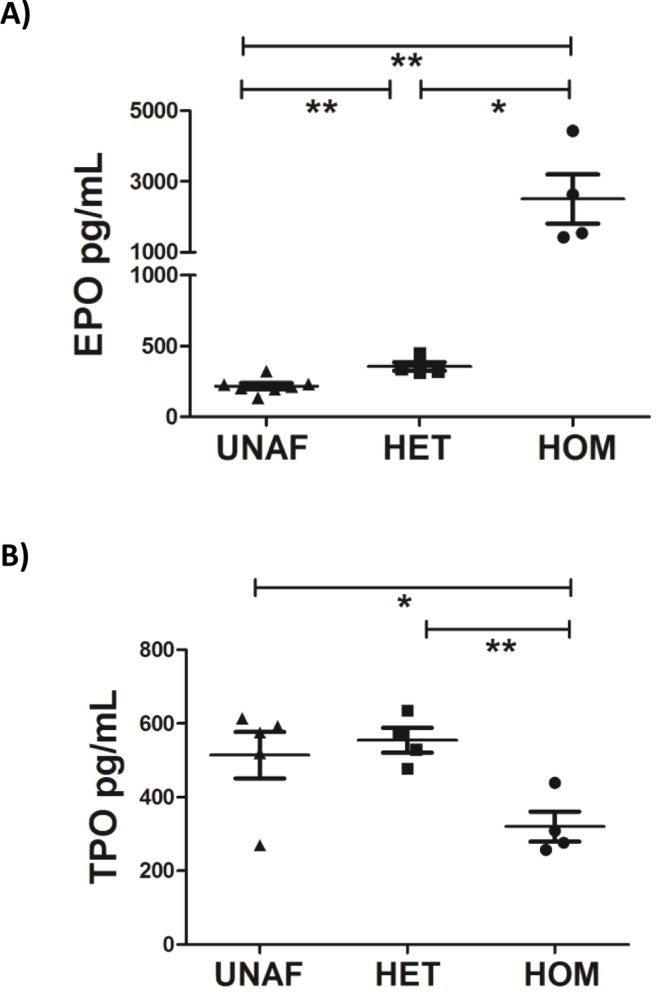
**Modulation of RBC and platelet growth factors in Ank1** ^**EX34**^ **pedigree. A)** RBC growth hormone EPO was significantly increased in both heterozygous (HET) and homozygous (HOM) Ank1^EX34^ mice. **B)** Megakaryocyte growth factor TPO was significantly decreased Ank1^EX34^ homozygote mice. *p < 0.05 ** p< 0.01 unpaired T-test *cf*. unaffected (UNAF).

Damaged or atypical RBCs are sequestered in the spleen and removed from circulation. Splenomegaly is a common feature in humans with HS and splenectomy is sometimes required [35]. Ank1^EX34^ mice were sacrificed and spleens excised to investigate abnormal RBC clearance. As expected, spleens from homozygous Ank1^EX34^ mice were enlarged, consistent with sequestration of abnormal RBC and/or increased RBC turnover (Figure 5). Increased RBC turnover and associated splenomegaly may lead to an inflammatory response. IL-6 and CCL5 (RANTES) were quantified in plasma of Ank1^EX34^ mice as an indication of inflammation. IL-6 was not detected in any of the samples assayed and there was no change in levels of CCL5 attributed to *Ank1* mutation (data not shown). EPO and TPO are key growth factors involved in RBC and platelet differentiation and haemostasis. EPO is a glycoprotein hormone that signals to increase RBC turnover and expression is increased in hypoxic conditions [39]. EPO levels were significantly increased in homozygous Ank1^EX34^ mice compared to heterozygotes and unaffected siblings (p = 0.021, p = 0.001 respectively, Figure 6A). Heterozygous Ank1^EX34^ mice also had significantly elevated EPO levels compared to unaffected siblings (p = 0.004, Figure 6A). TPO is a humoral growth factor that is necessary for megakaryocyte proliferation and maturation, as well as for thrombopoiesis [40]. Homozygous Ank1^EX34^ mice had significantly reduced TPO levels compared to unaffected siblings (p = 0.04, Figure 6B). The reduction in circulating TPO in homozygous Ank1^EX34^ mice is likely due to depletion via c-Mpl receptor binding consistent with the elevated platelet count in this pedigree.

## Discussion

Murine RBCs provide a suitable platform to study genetically defined changes in RBCs that may impact on structural integrity, differentiation and lifespan. These features are central to RBC functionality and any changes or disruption in RBC parameters may impact on the storage capacity of the blood component, and further may result in poor patient outcomes post-transfusion. Post-collection packed red blood cells are stored for up to 42 days before transfusion. Clinical studies have reported an association between RBC storage and increased patient morbidity post-transfusion [3–5], and there is evidence of donor variability in the lifespan of RBC in storage and onset of the storage lesion [6]. In this study, we used murine ENU models with both forward and reverse genetic approaches to study genetic-based changes in RBC parameters that may have important implications for storage of blood components for clinical use and transfusion outcomes in humans.

For the reverse genetics approach ENU pedigrees were chosen for investigation based on mutation in RBC structural proteins. At the commencement of the study, there were four defined mutations in genes encoding RBC structural proteins available for study; one mutation in *Epb4.1* (encoding band 4.1) and three separate mutations in *Spnb1* (encoding spectrin-1β). Band 4.1 and spectrin-1β are functionally important RBC cytoskeleton proteins. Band 4.1 associates with a number of RBC structural proteins including spectrin, actin, adducin, dematin, tropomodulin, tropomyosin, band 3 and glycophorin C in the 4.1R complex which spans the lipid bilayer [41]. Spectrin-1β is an intracellular self-assembling protein that in association with spectrin-1α forms an anchoring point for the band 3 and band 4.1R complexes. Thus both band 4.1 and spectrin-1β are key structural proteins that contribute to the stability and elasticity of the RBC. Mutations in these proteins have the potential to affect RBC structural integrity, differentiation and survival.

All of the SNVs studied in the spectrin-1β pedigrees occurred in the region encoding the spectrin-binding domain of the protein. It is not surprising that ENU mutagenesis would result in a number of mutations in this region as a large portion of *Spnb1* encodes the spectrin-binding domain. Of the three *Spnb1* SNVs studied, *Spnb1*(c) had the highest prediction of loss of function based on bioinformatic modelling. However, this mutation had the least impact of the three *Spnb1* mutations studied, with the pedigree exhibiting no evidence of altered haematological profile, and no difference in RBC structural integrity in terms of osmotic fragility or E5M binding. The spectrin-1β (a) and (b) pedigrees were both characterised by significantly increased RBC count, Hb and HCT, despite the low PolyPhen score and prediction of a benign mutation. Although there were increases in the RBC count (and associated Hb and MCV) in the spectrin-1β (a) and (b) pedigrees, the RBC did not appear to have altered structural integrity as no changes were apparent in osmotic fragility or E5M binding.

The SNV in *Epb4.1* results in a Thr→Ala amino acid substitution in the hydrophilic domain of the encoded band 4.1 protein and bioinformatic modelling determined this mutation was “probably damaging”. Despite the PolyPhen score and predicted disruption, we saw no evidence of changed blood picture due to mutation in *Epb4.1*. We observed a consistently reduced MCV and HCT in this pedigree, regardless of the presence or absence of the defined mutation in *Epb4.1*. ENU mutagenesis results in multiple mutations in the murine germ line and the reduced MCV and HCT is likely the result of a dominant mutation in another gene. Retrospectively, we assessed the other mutations reported to be present in the band 4.1 pedigree. There were 46 other SNV identified and the majority of the mutations were in genes primarily associated with cancer, cell cycle/survival and development and cardiovascular disease.

Of the 46 additional SNVs in the band 4.1 pedigree, on the basis of currently described function, mutations in *Trank1*, *E2f8*, *Etnk2* and *Yars2* will be the first investigated to determine if these mutations are associated with the reduced MCV and HCT in this pedigree. *Trank1* encodes tetratricopeptide repeat and ankyrin repeat-containing protein 1, a protein predicted to contain multiple ankyrin repeat domains. To date, there are limited reports on the function of this gene product, however, the presence of numerous ankyrin repeats and predicted “probably damaging” PolyPhen score as a result of a Phe→Leu amino acid substitution suggests SNV in *Trank1* has the potential to impact on RBC structural integrity. *E2f8* encodes E2f transcription factor 8. This transcription factor is also not well described however putative roles in cell cycle, endocycle and placental development have been reported [42]. Dysfunction in *E2f8* has been linked to anaemia [43]. The mutation in *E2f8* is predicted to be “possibly damaging” as a result of Ser→Ile amino acid substitution. Mutations in *Etnk2* and *Yars2* were also predicted to be “possibly damaging”. *Etnk2* encodes ethanolamine kinase 2, a member of the choline/ethanolamine kinase family. This enzyme catalyses the first step of phophatidylethanolamine biosynthesis and deficiency has been associated with altered haematologic parameters in mice [44]. *Yars2* encodes tyrosyl-tRNA synthetase 2, a mitochondrial protein that catalyses the attachment of tyrosine to tRNA. Mutations in this gene are associated with myopathy, lactic acidosis and sideroblastic anaemia type 2 [45]. Based on the presentation of the phenotype in all band 4.1 mice regardless of *Epb4.1* SNV carriage, we hypothesise an autosomal-dominant inheritance and matings for this pedigree are being designed to assist in the analysis of the causative mutation.

Using the forward genetic approach a novel ENU-mutant pedigree with an elevated reticulocyte count was identified. Further analysis revealed reduced Hb and lower MCV. Sequencing revealed a G→A substitution on Chromosome 8 (base pair position 23,119,434: GRCm38); the first base of the splice donor site 5’ of *Ank1* exon 34. The *Ank1* SNV occurs in a splice site motif in ankyrin-1 likely to be associated with loss of the exon 34 amino acid sequence. Ankyrin-1 is an erythrocyte cytoskeletal protein that provides a link between RBC membrane structural proteins (spectrin and band 3) and the inner surface of the lipid bilayer [14, 30]. Mutations in *Ank1* have been associated with hereditary spherocytosis (HS), an inherited haemolytic anaemia characterised by rigid spheroid RBCs [35]. Patients with spherocytosis exhibit anaemia with a compensatory reticulocytosis as a result of haemolysis of spherocytic red cells, jaundice, gallstones and splenomegaly [35].

The *Ank1* SNV reported in this study was different to previously reported *Ank1* mutations in both humans [15, 31] and mice [19, 32–34], although the phenotypes were similar. The Ank1^EX34^ pedigree characterised in this study demonstrates elevated reticulocyte count, reduced Hb, lower MCV, splenomegaly, reduced RBC E5M staining and increased RBC fragility, all of which are characteristic of HS. The Ank1^EX34^ pedigree also exhibited thrombocytosis, a characteristic not commonly associated with *Ank1* mutation or HS. *Ank1* mutations have also been associated with increased malarial resistance in a number of murine studies [34], and human HS as a result of *Ank1* mutation may be the result of evolution to prevent malarial infection. While malarial resistance was not the focus of our study, it is a reasonable hypothesis that the Ank1^EX34^ pedigree would demonstrate a resistance to malarial infection.

To further characterise this novel Ank1^EX34^ pedigree, we also studied potential changes in inflammation and modulation of RBC and platelet growth factors. Despite an altered haematological phenotype and splenomegaly in the Ank1^EX34^ pedigree, we did not find evidence of changes in the inflammatory mediators we studied. IL-6 is a pro-inflammatory cytokine, primarily synthesised by the liver. IL-6 is a pleiotropic cytokine with key roles in infection, inflammation, autoimmunity and tissue haemostasis [46]. IL-6 levels were at the limit of detection (<10pg/ml) in the Ank1^EX34^ pedigree, despite the clear dysfunction in a number of key biological systems in these mice. We also studied EPO and TPO as two key cytokines that contribute to RBC and platelet differentiation and haemostasis. EPO was significantly increased in both heterozygous and homozygous Ank1^EX34^ mice. EPO synthesis is increased in response to hypoxia and signals erythroid progenitor cells to differentiate into erythroblasts [39]. As the Ank1^EX344^ pedigree was defined in the first instance by an elevated reticulocyte count, it was not surprising that a significantly elevated EPO was associated with this phenotype. TPO is a humoral growth factor that is necessary for megakaryocyte proliferation and maturation, as well as for thrombopoiesis [40]. We observed significantly reduced TPO in homozygous Ank1^EX34^ mice. As these mice also exhibit thrombocytosis, it is likely the reduction in circulating TPO is due to depletion via c-Mpl receptor binding on platelets, though other mechanisms may also contribute to the TPO feedback loop, as high platelet count does not always correlate with reduced levels of TPO in the circulation [47, 48].

Using murine genetic models derived by ENU mutagenesis, RBC structural proteins were characterised in five pedigrees. Using a forward genetic screening approach we identified a phenotype characterised by reticulocytosis, microcytic anaemia and thrombocytosis. This phenotype was determined to be associated with a novel mutation in *Ank1*, encoding the RBC structural protein ankyrin-1. The forward approach was therefore a useful tool to identify and characterise the genetic basis of an altered haematological phenotype. As the Ank1^EX34^ mutation is in a splice motif, there is no PolyPhen score associated with this SNV. Four pedigrees were investigated from a reverse genetics approach, to identify a phenotype associated with the presence of a known mutation. For the SNVs studied we found the PolyPhen score was not correlated with a changed phenotype for any of the assays employed. There was no evidence of altered haematological parameters or RBC structural integrity resulting from the two SNVs that were predicted to result in a damaging phenotype (*Spnb1*(c) and *Epb4.1*). These mutations may be damaging, however altered phenotype may not have been evident due to molecular redundancy. The two SNVs predicted to be benign (*Spnb1* (a) and (b)) demonstrated an increased RBC count, HCT and Hb. Our findings suggest that new generations of bioinformatic prediction tools used in concert with feedback from phenotypic analysis will improve capacity to predict altered function.

This study was established in the context of understanding factors that may influence the outcomes of blood transfusion. The haemoglobin increment following transfusion is highly variable and there are minimal studies of non-serological variables associated with differences in post-transfusion survival of RBC. In this study we targeted SNVs in genes encoding proteins critical for RBC function that are expected to be important in post-transfusion RBC survival. The *Ank1* mutation is clearly associated with anaemia and elevated RBC turnover. The impact of the *Spnb1* mutations, while resulting in statistically significant increases in Hb does not produce profound changes that could be viewed as a disease, however they may be associated with altered post-transfusion RBC lifespan. These murine models provide a tool to study the contribution of genetic change to donor variability in blood component storage and post-transfusion RBC survival that may be associated with transfusion outcomes.
